# Changes in Antibiotic-Resistance Genes Induced by the Grazing Effect in Three Cladoceran Species

**DOI:** 10.3390/microorganisms9091959

**Published:** 2021-09-15

**Authors:** Jong-Yun Choi, Seong-Ki Kim

**Affiliations:** National Institute of Ecology, Seo-Cheon Gun 325-813, Korea; skkim@nie.re.kr

**Keywords:** *Daphnia*, tetracycline, wetland, antibiotic-resistant bacteria (ARB), microbiome

## Abstract

The acquisition of Antibiotic-Resistance Genes (ARGs) by natural bacteria caused by antibiotic abuse is causing serious problems for human and animal welfare. Here, we evaluated the influence of three cladoceran species on Antibiotic-Resistant Bacteria (ARB) and tetracycline-resistance gene (*tet*(A)) copies, and discussed the effect of these biological interactions on the distribution and diffusion of ARGs in freshwater ecosystems. Bacterial community and *tet*(A) abundances in water samples collected from wetlands were strongly influenced by cladoceran presence. The presence of *Daphnia obtusa* dramatically decreased ARB and *tet*(A) abundance compared to that with other cladoceran species (*Chydorus sphaericus* and *Simocephalus vetulus*). Interestingly, we found a high abundance of Flavobacteriales in the microbiomes of cladoceran species. Considering that Flavobacteriales species are potential carriers of the *tet*(A) gene, their adsorption and assimilation with cladocerans could significantly impact the reduction of *tet*(A) in water. Field surveys also showed that *tet*(A) abundance could be low if the dominance of *D. obtusa* in each wetland was high. This study highlighted the need for ecological interactions and a broad range of niches in the food web when discussing the fate of ARGs in freshwater ecosystems.

## 1. Introduction

The emergence of Antibiotic-Resistant Bacteria (ARB) is a major threat to human health and welfare at a time when antibiotic use continues to increase globally [1]. Infection of humans and animals by ARB is considered a serious problem, as antibiotic treatments are less effective [2]. Continued antibiotic use over the past 70 years has facilitated the acquisition of Antibiotic-Resistance Genes (ARGs) by natural bacteria distributed in various environments [3]. In general, chemical pollutants gradually decrease in concentration in water through processes such as decomposition, dilution, and absorption, but ARGs can continuously diffuse through gene transfer between bacteria in the environment. The abundance and spread of ARGs in various environments can be interpreted for a variety of reasons, but it can be mainly attributed to factors such as: (1) the horizontal gene transfer of ARGs between bacterial species or communities; (2) mutation and gene recombination; and (3) diffusion by selective pressure [4]. Although it is difficult to clearly understand the evolution and function of ARGs in the bacterial community because they are influenced by the complex effects of various environmental factors [4], efforts are needed to accurately identify and ease the spread of ARGs.

Freshwater ecosystems, such as rivers, lakes, and wetlands, are recognized as hotspots that facilitate the spread of ARGs [5,6]. These ecosystems are channels for the collection of various pollutants in the terrestrial environment, making it easier for antibiotics to enter and accumulate [7]. In particular, emissions from wastewater-treatment plants have been identified as the main reason for the increase in the acquisition of ARGs in the bacterial community [8]. The spread and accumulation of ARGs in freshwater ecosystems increase the risk of infection by resistant pathogens. Since freshwater ecosystems are not only used as habitats for a wide variety of wildlife, but are also closely related to human society (e.g., living space, drinking-water sources, and recreation; [9]), the diffusion of ARGs can be significant and bring potential threats. Thus, previous studies have suggested that freshwater environments serve as storage for the spread of ARGs, which can continue independent of human antibiotic use [10,11]. The continued spread of ARGs into freshwater ecosystems can induce a variety of ARG pools in the microbiomes of natural bacteria, leading to the transmission of genes to new and well-known pathogens [12]. Maintaining high concentrations of ARBs that are resistant to cephalothin, penicillin, tetracycline, ampicillin, and chloramphenicol in freshwater ecosystems means that not only are antibiotics continuously flowing into rivers, lakes, and wetlands, but so are ARB [6].

Recently, various empirical studies have quantitatively evaluated the presence of ARGs in freshwater ecosystems by applying a metagenomic approach, such as real-time PCR (qPCR) techniques [12,13,14]. Although these studies assessed the types and concentrations of ARGs in freshwater ecosystems, the knowledge of their spread and persistence remains limited. It is unclear what mechanisms and routes of ARG spread can be maintained continuously within the freshwater ecosystem. Whereas most empirical studies have focused on horizontal ARG transfer [15,16], vertical gene diffusion (i.e., trophic transmission of ARB and subsequent horizontal transfer of ARGs) cannot be ruled out [17]. However, gene transfer of ARGs through vertical pathways, such as biological interactions, is relatively less known. In this study, we focused on the gene transfer of ARGs between bacteria and cladocerans. Cladoceran communities are primary consumers commonly distributed in various freshwater ecosystems that mainly consume bacteria and phytoplankton [18,19]. Furthermore, considering that cladocerans are frequently used as food sources for top predators such as fish and macroinvertebrates, the continuous accumulation of ARGs induced by cladocerans can be an important mediator of ARG proliferation and vertical transmission in freshwater environments. Therefore, studies on the consumption by and in vivo accumulation of ARB in cladocerans are suitable for assessing the distribution and vertical transfer mechanisms of ARGs in freshwater ecosystems.

In this study, we evaluated the possibility that the continuous consumption of ARB by cladocerans can change the abundance of the tetracycline resistance gene *tet*(A) in water. It is important to study their distribution and spread in freshwater ecosystems because the *tet*(A) gene is most abundant in rivers, lakes, and wetlands, and is easily accumulated in various biological communities, such as invertebrates and fish. Previous studies have also suggested the possibility regarding bioaccumulation of these ARGs [6]. The potential attachment and adsorption of bacteria to the outer and internal surfaces based on the food consumption and continuous activity of cladocerans are expected to contribute to the reduction of the *tet*(A) gene in water. The bacterial community is a potential mediator of *tet*(A) spread, and the continuous consumption of ARB by cladocerans can contribute to a reduction in ARGs. The aim of this study was to elucidate the following: (1) the effects of ARB and ARGs mediated by different consumption by three cladoceran species; and (2) the influence of cladoceran species compositions on *tet*(A) gene abundances in wetlands. We evaluated gradual changes in bacteria and *tet*(A) 3 days later by providing field water rich in ARB and ARGs to three species of cladocerans (*Chydorus sphaericus*, *Simocephalus vetulus*, and *Daphnia obtusa*), which are frequently observed in freshwater ecosystems in South Korea. Due to the different bacterial consumption patterns among the three species of cladocerans, the species composition of cladocerans in various freshwater environments is believed to affect the spread and sustainability of ARB and ARGs.

## 2. Materials and Methods

### 2.1. Cladoceran Subculture and Experimental Design

The plankton culture was based on three cladoceran species: *C. sphaericus*, *S. vetulus*, and *D. obtusa*. These three species of cladocerans were collected from Upo Wetland, South Korea, in May 2019 and were maintained in the laboratory for over 5 months. The cladoceran species were preserved in 500 mL transparent glass beakers containing Elendt M4 medium [20] and were stored in a growth chamber (Eyela FLI-2000, Eyela, Tokyo, Japan) at 20 °C, with a 12L:12D light–dark cycle, with a 30-photon flux density (µmol∙m^−2^∙s^−1^). We provided the phytoplankton *Chlorella vulgaris* as a food source for cladocerans during the subculture maintenance period.

We chose approximately 100 offspring of each cladoceran species born within 24 h with similar life-history traits (e.g., birth time and size) from the stock culture and allowed them to reach the offspring-production stage. To obtain a similar-sized cohort, we first sorted and eliminated extraordinarily larger or smaller maternal cladoceran species from the sampled culture to finally obtain 50 adult cladocerans. The cladoceran individuals in the sample were cultured by adapting a scaled loupe (unit: mm). We transferred the 50 adult cladocerans to a new beaker filled with fresh Elendt M4 medium and provided sufficient food (*C. vulgaris*) until offspring were produced. To determine the quantity of food algae to provide, we considered the supply level appropriate for zooplankton population growth. A previous study suggested that an algal carbon content of approximately 2.5 mg C L^−1^ (units shown as mg C L^−1^ hereafter) in a given volume of zooplankton medium would be sufficient for zooplankton survival and population growth [21]. The first reproduction event occurred 3–4 days after selection. However, the number of neonates from the first reproduction cycle was small, and we used the second clutch from each individual cladoceran selected for the experiment. In summary, the initially selected cladoceran adults were used to produce the offspring employed in the main experimental procedure. The offspring from the second clutch were collected after birth (within 5 h) and used for the main experiment.

We designed four experimental groups to identify the different grazing effects of cladoceran species as follows: (1) without grazing by cladocerans; (2) water from the *C. sphaericus*-enriched treatment; (3) water from the *S. vetulus*-enriched treatment; and (4) water from the *D. obtusa*-enriched treatment. For each group, we prepared 20 replicates in 500 mL sterilized beakers filled with 500 mL field water (total: 80 beakers), and five acclimated individuals of each cladoceran were placed in beakers of each experimental group. Before placing each cladoceran individual into the experimental group, they were placed in Elendt M4 medium that contained no food for approximately 5–6 h to induce starvation. This is a measure to minimize the factors that might affect the grazing of each cladoceran species on bacteria. The field water samples collected from the Upo Wetlands located in South Korea were filtered using through 0.45 µm mixed cellulose ester membrane filters (A045A047A; Advantech Co. Ltd., Taipei, Taiwan) to remove large particles and metazoans. Each experimental group was maintained in a growth chamber (Eyela FLI-2000, Eyela, Tokyo, Japan) at 20 °C, with a 12L:12D light–dark cycle and a 30-photon flux density (µmol∙m^−2^∙s^−1^). Food algae were supplied at a quantity sufficient to maintain 2.5 mg C L^−1^ in each beaker throughout the experiment. The experiments were conducted for 3 days.

After completing the experiment (after 3 days), water samples from each beaker (total: 80 beakers; 20 replicates per group) of the four experimental groups were used for bacterial counting and DNA extraction. We also utilized the water samples before the experiment to compare the abundances of bacteria and *tet*(A) among the four experimental groups after the experiment. To compare the changes in the four experimental groups after 3 days of the initial experimental stage, we extracted a randomly selected initial water sample from 20 beakers in each experimental group for bacterial counting and DNA extraction. For cell counting of the bacterial community, we collected 1 mL of water samples from beakers in each experimental group and fixed the samples with formaldehyde (1.5% concentration). The water samples were stained with 1% SYBR Green, and bacterial abundances were determined using an Accuri C6 flow cytometer and its software (BD Biosciences). The remaining water sample in each beaker was then filtered through 0.22 µm polycarbonate filters and stored at −20 °C until molecular processing. Furthermore, for bacterial counting and DNA extraction of each cladoceran species, we isolated the cladoceran species injected into each beaker in three experimental groups after 3 days. Each was stored separately in glass vials filled with 60% ethanol to prevent cross-contamination among individuals before DNA extraction to eliminate extracellular DNA attached to the exoskeleton. The exoskeleton of each individual was exposed to commercial bleach diluted 2.5% for 2 min and then washed three times with distilled water to avoid any effects from the remaining bleach on the skeleton [22]. Pretreated cladoceran individuals were stored individually in 2 mL microtubes (total: 80 samples).

We designed additional experiments to understand the relationship between *D. obtusa* density and *tet*(A) abundance. Previous experiments have shown that *D. obtusa* has a dramatic effect on the reduction in ARGs (i.e., *tet*(A)) among the three cladoceran species. For this experiment, *D. obtusa* was used with a total of 10 experimental groups, divided per 10 individuals in a range of at least 10 to 100, and the *D. obtusa* individuals were those used in the previous experiment (the offspring from the second clutch were collected after birth). To minimize the error based on the results from each experimental group, we prepared 10 replicates for each experimental group. Each experiment was conducted in a tank with an acceptable size with up to 100 individuals (approximately 27 L in total volume; 30 × 30 × 30 cm). Water samples in each tank collected from the Upo Wetlands located in South Korea were filtered using through 0.45 µm mixed cellulose ester membrane filters (A045A047A; Advantech Co. Ltd., Taipei, Taiwan) to remove large particles and metazoans. Each experimental group was maintained for 3 days after *D. obtusa* injection, and after the experiment, water samples in the tank were collected for an analysis of *tet*(A).

### 2.2. Field Survey

Additional field surveys were conducted to understand the influence of cladoceran species composition on ARG abundance in aquatic ecosystems. We investigated the cladoceran community and *tet*(A) gene in 20 riverine wetlands located in the mid-lower reaches of the Nakdong River from May to June 2019. Among the 40 wetlands studied by Choi and Kim [19], wetlands 1–20, where cladocerans were abundant, were selected as the survey site. Among the 11 L water samples collected from each wetland, 10 L were used for the quantification of cladoceran individuals, and the remaining 1 L was used to analyze the ARG. The 10 L of wetland water was filtered using a plankton net (32 μm mesh), and the filtrate was preserved in sugar formalin (final concentration: 4% for formaldehyde; [23]). Cladoceran enumeration and identification at the genus level were performed using a microscope (Model Axioskop 40; Carl Zeiss; Oberkochen, Germany; 200× magnification), with identification based on the classification key published by Mizuno and Takahachi [24].

### 2.3. DNA Extraction and Gene Detection

#### 2.3.1. Bacterial Abundance

In each experimental group, water samples and cladoceran individuals were processed separately for DNA extraction to identify bacterial abundance and the ARG (i.e., *tet*(A)). First, for bacterial abundance in each sample, genomic DNA was obtained using a DNeasy Blood and Tissue Kit (Qiagen, Hilden, Germany) following the manufacturer’s instructions, except for the following differences: (1) twice the amount of buffer ATL (360 µL) and proteinase K (40 µL) was used on the filter paper samples to increase DNA extraction; (2) the DNA elution step was repeated; and (3) the final amount of DNA extracted from each sample was 50 µL. The extracted DNA was stored at −20 °C.

Extracted DNA for sequencing was prepared according to Illumina 16S metagenomic sequencing library protocols (San Diego, CA, USA). DNA quantity, quality, and integrity were measured using PicoGreen (Thermo Fisher Scientific, Waltham, MA, USA) and a VICTOR Nivo Multimode Microplate Reader (PerkinElmer, Waltham, MA, USA). Amplification was performed using AccuPower Hot Start PCR PreMix (Bioneer, Korea) with genomic DNA and primers in a final volume of 20 µL. We used bacterial primers targeting the V3/V4 region of the 16S rRNA, including an adapter sequence for Illumina, as follows: forward, 50-TCGTCGGCAGCGTCAGATGTGTATAAGAGACAGCCTACGGGNGGCWGCAG-30; and reverse, 50-GTCTCGTGGGCTCGGAGATGTGTATAAGAGACAGGACTACHVGGGATCTAATCC-30 [25]. Gradient PCR was performed using a thermal cycler (Bio-Rad, Hercules, CA, USA) under the following conditions: initial denaturation at 95 °C for 3 min, followed by 30 cycles of denaturation at 95 °C for 30 s, annealing at 55–65 °C for 30 s, elongation at 72 °C for 30 s, and a final extension at 72 °C for 5 min. After extension, the reaction was performed at 4 °C. Amplification products were separated using 1.5% gel electrophoresis.

After amplification, to generate indexing PCR products, the PCR product was amplified with one cycle of 3 min at 95 °C, eight cycles of 30 s at 95 °C, 30 s at 55 °C, 30 s at 72 °C, and a final step of 5 min at 72 °C. A subsequent limited-cycle amplification step was performed to add multiplexing indices. The final products were normalized and pooled using PicoGreen (Thermo Fisher Scientific, Waltham, MA, USA), and the sizes of libraries were verified using the LabChip GX HT DNA High Sensitivity Kit (PerkinElmer, Waltham, MA, USA); NGS analysis, including index PCR, was completed by Macrogen Co. (Seoul, Korea). The sequencing library was prepared by random fragmentation of the DNA or cDNA sample, followed by 5′ and 3′ adapter ligation. Alternatively, “tagmentation” combined the fragmentation and ligation reactions into a single step that greatly increased the efficiency of the library-preparation process. Adapter-ligated fragments were then PCR-amplified and gel-purified. The PCR products were sequenced using the MiSeq™ platform (Illumina, San Diego, CA, USA) from a commercial service (Macrogen Inc., Seoul, Korea).

Raw reads were trimmed with CD-HIT-OTU, and chimeras were identified and removed using rDNA Tools. For paired-end merging, Fast Length Adjustment of Short reads (FLASH) version 1.2.11 was used. Merged reads were processed and clustered into OTUs using the bioinformatics algorithm UCLUST [26] at a 97% OTU cut-off value (352 OTUs in gamma-diversity). Taxonomy was assigned to the representative sequences obtained using BLAST (Reference DB: NCBI—18S) [27] using UCLUST [26]. For the aforementioned processes of BLAST and UCLUST, we used an open-source bioinformatics pipeline to perform microbiome analysis, namely QIIME version 2 [28]. We classified each OTU according to identity percentage (%) as follows: species level ≥97%; genus level ≥90%; and family level with ≥84% reads with less than 84% identity were excluded.

#### 2.3.2. ARG

DNA was extracted from the remaining water samples using a commercial kit following the manufacturer’s protocol (Ultra Clean Microbial DNA Isolation Kit, MoBio Laboratories). The ARG (*tet*(A)) was detected by PCR using the MyCycler thermocycler (Bio-Rad). The ARG was identified from a total of three samples, specifically: (1) water samples; (2) cladoceran individuals of each experimental group; and (3) water samples collected from the field survey. For the analysis of ARGs, we followed two steps as follows. First, we tried to amplify the resistance genes in all samples with normal PCR assays. If the gene was amplified in any sample; that is, if there was only one visible band, qPCR assays were established for this gene using the same primers. All PCR assays were performed in a volume of 25 µL containing 2 µL of DNA (diluted 1:20), 0.5 µM of each primer, and 12.5 µL of Go-Taq Green Master Mix (Promega). The PCR program was as follows: 95 °C for 3 min, 30 cycles of 95 °C for 30 s, annealing temperature (AT) specific for each gene and primer pair (Table 1) for 1 min, 72 °C for 30 s, and a final extension at 72 °C for 7 min.

Quantitative real-time PCR assays were then established for the *tet*(A) genes that resulted in positive PCR amplification. They were quantified by qPCR using the RT-thermocycler CFX Connect (Bio-Rad). Standard calibration curves were generated using the purified, quantified, and 10-fold-diluted amplicon of each gene as described by Di Cesare et al. [29], with the following minor modifications: the concentration of the stock solution of each gene was quantified with a GloMax (Promega) spectro-fluorimeter plate reader; and qPCRs were performed in a 20 µL volume with 2 µL of DNA (diluted 1:2), 0.5 µM of each primer, 10 µL of SsoAdvanced universal SYBR Green supermix (Bio-Rad), and filtered and autoclaved MilliQ water (Millipore) to the final volume.

The qPCR program was 95 °C for 2 min, 35 cycles of 95 °C for 15 s, AT (Table 1) for 30 s, and 72 °C for 15 s. Melt-curve analysis was performed from 60 to 95 °C with increments of 0.5 °C/5 s. Each reaction was carried out in duplicate for each sample. To ensure the specificity of the qPCR, the specific melting peak was confirmed using the PRECISION MELT ANALYSIS Software 1.2, built in to CFX ANAGER Software 3.1 (Bio-Rad), and all qPCR products, including the standards, were visualized by electrophoresis (30 min at 80 V, 1.5% agarose gel). The limit of detection (LOD) of the standard curves was determined as described by Bustin et al. [30]. In brief, the LOD for each gene was the minimum concentration that was detected and was in accordance with the linearity of the standard curve for all runs necessary to quantify each gene (Table 1). The reaction efficiency (always between 98.3% and 120.8%) and the regression coefficient (R^2^, always >0.96) were determined.

For samples in which the threshold cycle was below the LOD but above the limit of the qPCR (theoretically three copies per PCR; Bustin et al. [30]), the analyzed gene was considered present but not quantifiable. The qPCR results expressed as per reaction were converted into copy numbers of the single genes as described in detail by Di Cesare et al. [29]. The average concentrations of the two replicates for each gene were calculated and used for subsequent analyses. For the samples in which one replicate was quantifiable and the other one was negative, the gene was considered positive but not quantifiable. In the case of one replicate that was quantifiable and one positive that was not quantifiable, we considered the sample as quantifiable. ARG abundances were expressed as ARG copies per mL of filtered water, relative to 16S rDNA gene copies [33,34].

## 3. Results

**Figure 1:**
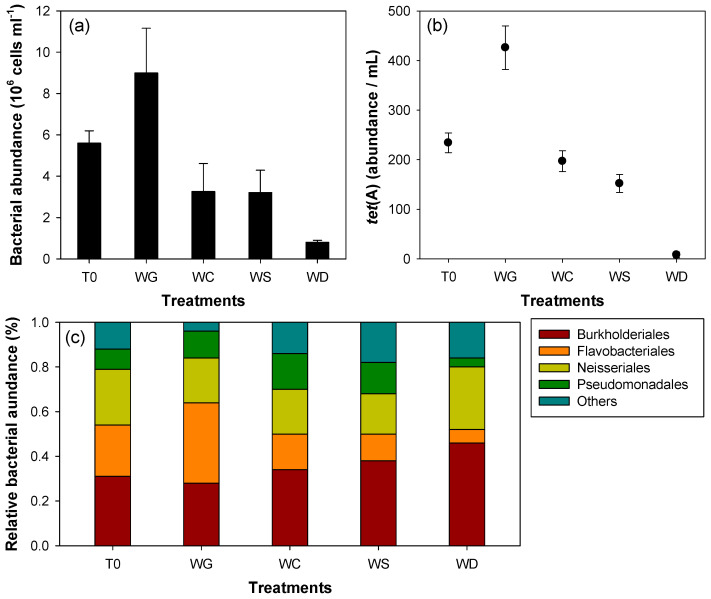
Bacterial abundance and *tet*(A) gene in water samples prior to treatment (T0) and in the four experiment groups (WG, WC, WS, and WD) after 3 days: (**a**) bacterial abundance; (**b**) *tet*(A) abundance per mL; and (**c**) the relative bacterial abundance (%). WG, without gazing by cladocerans; WC, water of the *Chydorus sphaericus*-enriched treatment; WS, water of the *Simocephalus vetulus*-enriched treatment; WD, water of the *Daphnia obtusa*-enriched treatment.

The bacterial and *tet*(A) abundances between each experimental group were clearly different (Figure 1a,b; one-way ANOVA, *p* < 0.05). The presence of cladocerans significantly reduced the abundance of bacteria and *tet*(A) compared to those with their absence. Among the experimental groups, the treatment administered with *D. obtusa* (water from the *D. obtusa*-enriched treatment, WD) contributed strongly to the reduction in bacteria and *tet*(A) abundances. *Chydorus sphaericus* and *S. vetulus* also reduced bacteria and *tet*(A) abundance by half, but these were higher than those with treatment administered with *D. obtusa*. In addition, the effect of reducing bacteria and *tet*(A) abundances was slightly different between the treatments administered with *C. sphaericus* and *S. vetulus* (WC and WS, respectively). The relative abundance of bacteria changed between each experimental group (Figure 1c). In particular, the relative abundance of Flavobacteriales decreased significantly after the administration of cladocerans compared to that at the beginning of the experiment. This reduction in Flavobacteriales was the highest in the treatment group administered WD, followed by that with WS and WC. In contrast, the relative abundance of Flavobacteriales in the treatment group not administered cladocerans (WG) was more than double that in the treatment group administered cladocerans.

**Figure 2:**
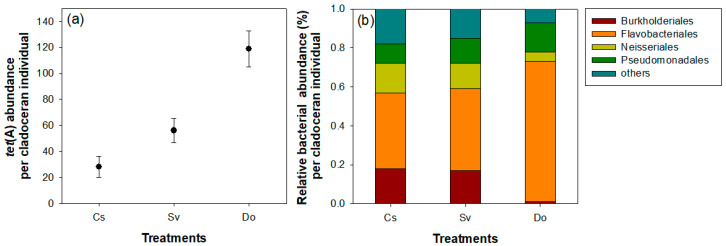
The *tet*(A) abundance (**a**) and relative bacterial abundance (%) (**b**) in each cladoceran individual. Cs, *Chydorus sphaericus*; Sv, *Simocephalus vetulus*; Do, *Daphnia obtusa*.

In each treatment group administered cladocerans, the relative abundances of *tet*(A) and bacteria in the body of each cladoceran differed among species (Figure 2). The *tet*(A) abundance in the body of *D. obtusa* was the highest among cladoceran species, whereas *tet*(A) abundance *in C. sphaericus* and *S. vetulus* was similar. Among the bacterial communities, the highest relative abundance of Flavobacteriales was also found (72%) in the body of *D. obtusa*, with the two remaining cladoceran species (*C. sphaericus* and *S. vetulus*) showing similar bacterial compositions.

**Figure 3:**
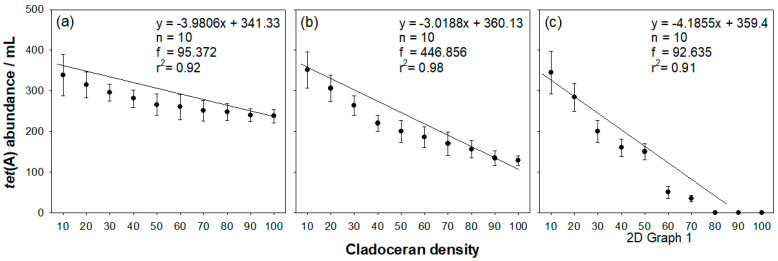
The relationship between cladoceran density and *tet*(A) abundance in water (regression df = 1, residual df = 8): (**a**) *Daphnia obtusa* and *tet*(A) abundance; (**b**) *Simocephalus vetulus* and *tet*(A) abundance; and (**c**) *Cydorus sphaericus* and *tet*(A) abundance.

The gradual increase in cladoceran density per water volume in the tank reduced ARG abundance (Figure 3). In addition, we demonstrated that there was a negative correlation between *tet*(A) gene abundance and the three species of cladocerans. *Daphnia obtusa* contributed more strongly to the decrease in *tet*(A) than the other two cladoceran species. When *D. obtusa* was administered at a number of more than 80 individuals, there were few *tet*(A) genes left in the tank after 3 days. In contrast, despite the fact that the maximum number of *S. vetulus* and *C. sphaericus* individuals were injected into the tank, the *tet*(A) gene was not observed to decrease more than that with *D. obtusa*. However, the two cladocerans and the *tet*(A) gene were also significantly different, such as with the results obtained for *D. obtusa*.

**Figure 4:**
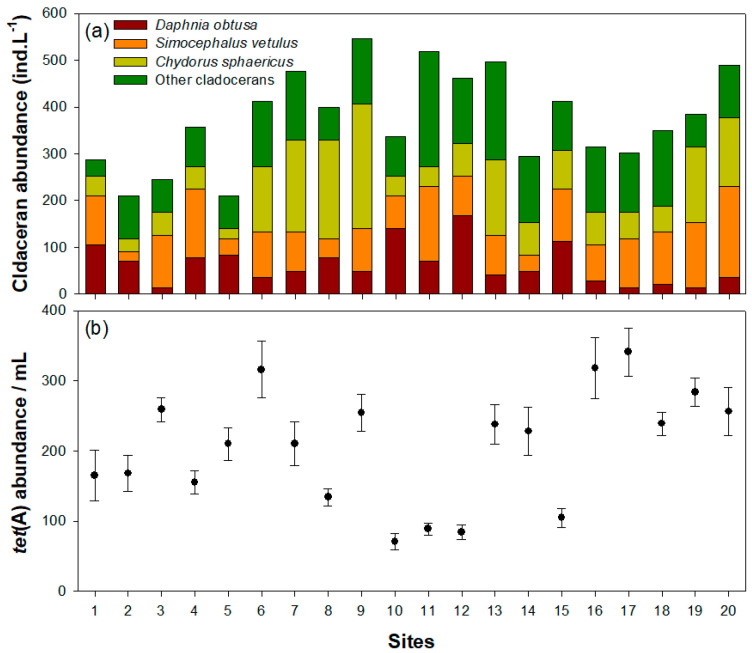
Cladoceran abundance and *tet*(A) abundance in 20 wetlands: (**a**) cladoceran abundance; (**b**) *tet*(A) abundance.

The species composition of cladocerans in each wetland affected the *tet*(A) abundance (Figure 4). The cladoceran community showed different species compositions and dominant patterns among 20 wetlands, which contributed to many and few *tet*(A) genes. Low abundance of *tet*(A) was mainly found in wetlands 1, 10, 12, and 15, where *D. obtusa* was found to be present at numbers of more than 100 individuals. The abundance of the *tet*(A) gene in each wetland decreased with an increasing density of *D. obtusa* (df = 18, F = 50.44, and r^2^ = 0.74). However, there have been few studies on the effect of the density of the other two cladoceran species on *tet*(A) abundance. However, in the aforementioned culture experiment, we found that *C. sphaericus* and *S. vetulus* contributed to the decrease in the abundance of the *tet*(A) gene in 20 wetland samples, and the *tet*(A) gene was maintained at a high abundance despite the dominance of these two species.

## 4. Discussion

In this study, we found that the consumption of bacterial communities by cladocerans decreased ARB abundance. The presence of cladocerans clearly reduced *tet*(A) abundance by approximately 75%. At the beginning of the experiment, the bacterial composition of water in beakers was similar to the bacterial composition in the microbiome of cladocerans after the administration of the cladocerans (a total of eight genera), and was dominated by Burkholderiales and Flavobacteriales. This means that cladocerans can be utilized by most bacterial communities in field water. The reducing effect of cladocerans on bacteria and *tet*(A) abundance differed depending on the species injected per experimental group. Interestingly, we found that the cladocerans clearly reduced Flavobacteriales abundances in the freshwater bacterial community. Prior to the input of cladocerans, the relative abundance of Flavobacteriales in each experimental group was approximately 23%, but this decreased to less than 15% after the administration of cladocerans (after 3 days). These results were clearly different from those of an experimental group (WG) that did not contain cladocerans. We found that the relative abundance of Flavobacteriales was approximately 36% after 3 days in the experimental group (WG) that did not receive cladocerans, an increase of 13% compared to that at the beginning of the experiment. Previous studies also suggested that Flavobacteriales can be dominant in wetlands or reservoirs where physical (such as rainfall and torrential currents) and biological (such as predation and competition) disturbances are small [35,36].

*Daphnia obtusa* had the strongest influence on the decline in the Flavobacteriales community and *tet*(A) genes compared to that with other cladoceran species (*C. sphaericus* and *S. vetulus*). Similarly, Eckert et al. [31] also suggested that the consumption of ARB by *D. obtusa* clearly decreased *tet*(A) abundance. *Daphnia obtusa* is a typical pelagic species that has a high bacteria and phytoplankton consumption pattern because they swim over a wide range through frequent movements [32,37]. From this point, we speculate that *D. obtusa* might have reduced bacterial abundance while swimming over the entire range in each beaker. Choi et al. [38] suggested that *C. sphaericus* and *S. vetulus* mainly consume algae attached to the leaves and roots of aquatic macrophytes, such as *Ceratophyllum demersum* and *Salvinia natans* in wetlands, whereas their contribution to pelagic food sources is relatively low due to their limited movement. Thus, *C. sphaericus* and *S. vetulus* consume only bacteria around their locations, which might have a relatively modest effect of reducing ARB and *tet*(A) abundance.

After 3 days (during the experimental period), we found a high abundance of Flavobacteriales among bacterial communities in the microbiomes of the three cladoceran species. In particular, the relative abundance of Flavobacteriales in *D. obtusa* was higher than that in *C. sphaericus* and *S. vetulus* (76%). However, *D. obtusa* did not appear to exhibit the selective consumption of Flavobacteriales among various bacterial communities. Food consumption in the cladoceran community, including *D. obtusa*, is typically via filter feeding, making it impossible to capture only one particular food source. Furthermore, the beakers or tanks used for the experiments neither allowed *D. obtusa* to swim around as freely as they would in nature, nor allowed the other cladocerans to feed benthically. The feeding behavior of all three cladocerans in this context may therefore be unnatural (or, at least, constrained based on how much of the algae is floating, suspended, or settled during the course of the experiments). This was an obvious limitation of this experiment. We considered that the high abundance of Flavobacteriales in the microbiome of *D. obtusa* was due to easy adsorption or a high assimilation rate on the gut surface of *D. obtusa*. Hall-Standly et al. [39] suggested that the species composition of bacteria attached to the skin or visceral surfaces of animals has similar structures to biofilms, depending on physical structures or morphological benefits in the gut. The high abundance of Flavobacteriales in the microbiome of *D. obtusa* is closely related to the decrease in *tet*(A) abundance. Empirical studies have reported that Flavobacteriales species are likely to be carriers that harbor high levels of *tet*(A) among bacterial communities [40,41]. Flavobacteriales species are abundant in freshwater ecosystems, as they comprise an opportunistically growing classification group and cause problems as fish pathogens. Although *C. sphaericus* and *S. vetulus* also contributed to the decrease in *tet*(A) abundance, their effects were lower than *those of D. obtusa*. From this finding, we determined that the relative abundance of Flavobacteriales in the microbiome of cladocerans has a close positive correlation with *tet*(A) abundance. Through further experiments, we found that an increase in the individual number of *D. obtusa* had a clear effect on reducing *tet*(A) abundance. This reducing effect was dramatic compared to that of *C. sphaericus* and *S. vetulus*. These additional experimental results provided conclusive evidence that the marked consumption of Flavobacteriales by cladocerans in previous beaker experiments reduced *tet*(A) abundance.

However, although the presence of cladocerans in this experiment significantly contributed to the reduction in ARB and *tet*(A) abundance in water, the presence of this ARG in the microbiota of cladocerans indicated that it potentially remained. While the ARG in the microbiota of cladocerans might be considered environmental DNA, in laboratory experiments, it is clear that this ARG was consumed by cladocerans, resulting in the increased number of ARG copies in the microbiota of the cladoceran species injected into each beaker after 3 days compared to that at the beginning of the experiment. The ARBs, including the *tet*(A) gene consumed by cladocerans, form part of *the D. obtusa* microbiome, which can contribute to the genetic abundance of a holobiont (genes of the animal and associated microbes [42]). If cladocerans with the *tet*(A) gene are consumed by fish or invertebrates, they will acquire the *tet*(A) gene [43]. ARGs, including *tet*(A), are likely to spread rapidly, because cladocerans can move significantly over long distances according to the water flow in freshwater ecosystems.

In 20 wetlands, the species composition of cladocerans clearly influenced *tet*(A) abundance in water. Although these results might suggest various factors affecting the *tet*(A) gene in water because of the field survey, the relationship between crustaceans and ARGs is not negligible because these wetlands have a small area of less than 1 km^2^. The results of these field surveys were consistent with those of the microcosm experiments. When *D. obtusa* was abundant in the wetlands, *tet*(A) gene abundance remained relatively low. In contrast, the *tet*(A) gene had very little relevance to other cladoceran species. However, previous studies have suggested that the negative effects of ARGs mediated by *D. obtusa* in freshwater ecosystems are difficult to predict. Choi and Kim [44] pointed out that frequent movements of pelagic cladocerans, including *D. obtusa*, make them easy prey for predators searching for food. As long as there is no refuge, such as aquatic macrophytes in freshwater ecosystems, *D. obtusa* numbers can be very low or absent due to the continuous foraging activity of fish on cladocerans [19,45]. In particular, it is more difficult for *D. obtusa* to inhabit areas when fish specialize in the predation of cladocerans, such as *Lepomis macrochirus in* Korea’s freshwater ecosystems [19]. Therefore, wetlands and reservoirs in South Korea are dominated by epiphytic cladoceran species, such as *C. sphaericus*. In such an environments, a decrease in ARB mediated by cladocerans would not be expected.

Among the various ecosystems, freshwater environments, such as rivers, lakes, and wetlands, have contributed greatly to the spread of ARGs. Empirical studies have suggested that the abundance of ARB in the total bacterial community is higher than that in other freshwater ecosystems, at 98% in rivers and 77% in lakes [4]. Lotic ecosystems such as rivers and lakes have a higher abundance of ARB than wetlands and ponds because they occupy relatively greater areas than other freshwater ecosystems. Since the area in contact with various regions, such as the terrestrial environment, is relatively large, diverse organisms, including bacterial communities, can be input. This research on interactions between cladocerans and other organisms in these environments can refocus on one of the many opportunities overlooked to date. Ecological, physiological, and genetic information related to cladocerans, including the consumption patterns of bacteria by cladocerans, has been sufficiently presented in previous studies, creating conditions for continuous studies on the relationship between cladocerans and ARB. The negative effect of ARB mediated by cladocerans can be utilized as a biological management method to prevent the spread of ARGs and reduce their abundance.

## Figures and Tables

**Table 1:** Primers pairs used to detect and/or quantify 16S rDNA and antibiotic-resistance genes.

Target Name	Primer Name	Primer Sequence (5′–3′)	Amplicon Size (bp)	Annealing Temperature (°C)	Reference
16SrDNA	Bact1369F	CGGTGAATACGTTCYCGG	142	55	Suzuki et al. [31]
	Prok1492R	GGHTACCTTGTTACGACTT			
*tet*(A)	*tet*(A)F	GCTACATCCTGCTTGCCTTC	210	64	Ng et al. [32]
	*tet*(A)R	CATAGATCGCCGTGAAGAGG			

## Data Availability

The data presented in this study are available upon request from the corresponding author. The data are not publicly available due to privacy restrictions.
